# Towards the Use of Individual Fluorescent Nanoparticles as Ratiometric Sensors: Spectral Robustness of Ultrabright Nanoporous Silica Nanoparticles

**DOI:** 10.3390/s23073471

**Published:** 2023-03-26

**Authors:** Mahshid Iraniparast, Berney Peng, Igor Sokolov

**Affiliations:** 1 Department of Mechanical Engineering, Tufts University, Medford, MA 02155, USA; 2 Department of Physics, Tufts University, Medford, MA 02155, USA

**Keywords:** fluorescent sensors, ratiometric sensors, nanosensors, sensing at the nanoscale, nanoparticles

## Abstract

Here we address an important roadblock that prevents the use of bright fluorescent nanoparticles as individual ratiometric sensors: the possible variation of fluorescence spectra between individual nanoparticles. Ratiometric measurements using florescent dyes have shown their utility in measuring the spatial distribution of temperature, acidity, and concentration of various ions. However, the dyes have a serious limitation in their use as sensors; namely, their fluorescent spectra can change due to interactions with the surrounding dye. Encapsulation of the d, e in a porous material can solve this issue. Recently, we demonstrated the use of ultrabright nanoporous silica nanoparticles (UNSNP) to measure temperature and acidity. The particles have at least two kinds of encapsulated dyes. Ultrahigh brightness of the particles allows measuring of the signal of interest at the single particle level. However, it raises the problem of spectral variation between particles, which is impossible to control at the nanoscale. Here, we study spectral variations between the UNSNP which have two different encapsulated dyes: rhodamine R6G and RB. The dyes can be used to measure temperature. We synthesized these particles using three different ratios of the dyes. We measured the spectra of individual nanoparticles and compared them with simulations. We observed a rather small variation of fluorescence spectra between individual UNSNP, and the spectra were in very good agreement with the results of our simulations. Thus, one can conclude that individual UNSNP can be used as effective ratiometric sensors.

## 1. Introduction

For many years, concentrations of ions and acidity were measured using macroscopic methods such as titration, litmus paper, or electrochemical cells. However, the spatial and temporal resolutions of those methods prevented the detection of ion concentrations within cells or tissues [1,2,3]. Measurements of temperature at the submicron scale were limited too. Sensors working at the nanoscale (nanosensors) can address this challenge, in particular, fluorescence-based nanosensors [4,5,6,7,8,9]. Nanosensors demonstrate high stability and brightness compared to other methods [10,11,12,13]. The architecture and composition of nanosensors can be tuned for bioanalytical applications [14,15,16]. To obtain quantitative measurements, ratiometric fluorescent nanosensors are a popular choice. In ratiometric fluorescent nanosensors, one part of the spectrum should respond to the stimuli differently from the other. The ratio of the intensities of these two spectral bands is independent of the excitation intensity, thereby giving a reliable quantitative reading of the properties of the surroundings. As an example, two different fluorescent dyes can be used for such a sensor. One dye is responsive to the stimulus, whereas the second dye serves as a reference [5,10]. The advantages of ratiometric nanosensors include the minimum effect by the fluctuation of the excitation source, the concentration of the sensor, and other drifts in the environment or the instrument [4,11,17].

One example of nanosensors is ultrabright fluorescent nanoparticle-based sensors. Ultrabright fluorescent silica nanoparticles (UNSNP) is a class of exceptionally bright particles in which fluorescent organic dyes are encapsulated inside a nanoporous silica matrix [18,19,20,21]. This approach allows the encapsulation of multiple dyes without their chemical modification [22,23]. Dyes after encapsulation exhibit excellent photostability. This effect is particularly strong for infrared dyes [24]. Multiple dyes can be encapsulated within the same particle [25,26]. The approach, initially demonstrated on micron-size particles, has been successfully extended to UNSNP [20,21,27]. Functionalization of UNSNP with biologically active molecules has been demonstrated for the successful detection of cervical cancer in vitro [28] and in vivo [12].

Fluorescent ultrabrightness comes from the ability of particles to accumulate a very large concentration of dye molecules without quenching their fluorescence [29]. This effect comes from a complex nanoscale environment of mesoporous silica which surrounds the dye molecules. The high concentration of the dye molecules leads to the proximity of the fluorescent dye molecules. As a result, the encapsulated dyes exhibit Förster resonance energy transfer (FRET), meaning that the excitation energy adsorbed by one dye molecule (donor) can be transferred to another dye molecule (acceptor) [25,26]. The approach used to create ultrabright fluorescent nanoparticles can be used to create complex fluorescent spectra of particles, beneficial in multiplexed biomedical imaging, flow cytometry, and sensors [11,26,30]. The ability to create sensors of temperature and acidity has been recently demonstrated [7].

Multiple research groups have investigated the encapsulation of multiple dyes inside the nanoporous silica matrix. Two different dyes were encapsulated in a silica framework, including an NIR heptamethine cyanine dye and a nonfluorescent naphthalocyanine dye. The resulting UNSNP could be used for bioimaging and therapeutic applications simultaneously [31]. A multi-dye ratiometric nanoparticle sensor based on sol-gel silica was developed to measure subcellular molecular oxygen [32]. Besides silica, ratiometric nanosensors can be made of polymers. For instance, a polymer-based ratiometric nanosensor for the detection of mercury ions in water [33]. Semiconductor polymer-based nanoparticles (Pdots) were also used as a platform for ratiometric sensors of acidity [17].

Dyes can be used in nanoparticles through physical adsorption, encapsulation, or covalent bonding. Covalent immobilization offers the most stable dyes [34]. However, covalent immobilization typically leads to a decrease in the quantum yield of dyes. Noncovalent encapsulation is a more universal approach since it does not require chemical modification of the dyes [14,29]. It can be utilized for sensing a broad range of stimuli. For example, probes encapsulated by biologically localized embedding (PEBBLEs) were used as nanosensors of a variety of ions, such as H^+^, Ca^2+^, Zn^2+^, Cu^2+^, Mg^2+^, or K^+^ [35].

Leakage of dyes is one of the issues that might occur in the noncovalent encapsulation of dyes. There are different approaches to prevent the potential leakage of dyes from silica pores. For example, a small number of hydrophobic groups were introduced to the silica matrix [20,27]. As was demonstrated, it eliminated the problem of dye leakage while keeping the surface of the particle hydrophilic [27,36]. The use of polystyrene as the polymer matrix prevented the leakage of dyes due to the strong hydrophobic interaction between the dye and the matrix [37].

In the present work, we address the major roadblock of using individual UNSNP as nano sensors: the uncontrollable ratio between encapsulated dye molecules of different kinds within each particle. To use these particles as sensors or as labels for multiplexing, it is paramount to show that the fluorescence spectra collected from individual nanoparticles are similar to each other. Due to fundamental thermodynamic fluctuations, such a ratio cannot be controlled during the synthesis of these particles. Secondly, dye molecules, which are randomly distributed inside each nanoparticle, can be arranged quite differently from particle to particle. It can lead to a substantially different interaction between dye molecules (mostly via FRET) and, consequently, to different fluorescent spectra.

Here, we study spectral variations using both direct measurements and simulations. The direct measurements were performed using a confocal fluorescent microscope equipped with a spectroscopic camera capable of measuring luminescence spectra at each image pixel. The measurements were compared with the results of simulations, in which the dyes were randomly distributed inside the particles. A very good agreement between the simulations and measurements was observed.

The observed variations of spectra were rather small. Converted to measuring temperature, for example, the variation (the standard deviation of the mean) was only 0.4 °C when using one particle and 0.1 °C when using the results of averaging over five particles (where the signal collection time for each spectrum was 30 ms). It is worth noting that the same dyes could be used to create a variety of distinguishable spectra for multiplexing applications [25,26].

## 2. Materials and Methods

### 2.1. Chemicals

Tetraethylorthosilicate (TEOS, ≥99%, GC, Acros Organics, Fair Lawn, NJ, USA), triethanolamine (TEA, reagent grade 98%, Sigma Aldrich, St. Louis, MO, USA), cetyltrimethylammonium bromide (CTAB, High Purity Grade, Amresco, Solon, OH, USA), ethyltriethoxysilane (ETES, 96%, Frontier Scientific, Logan, UT, USA), rhodamine 6G (R6G, Sigma Aldrich), and rhodamine B (RB, Exciton, Dayton, OH, USA) were used. RC membrane (RC membrane, Spectra/Pore, Rancho Daminguez, CA, USA) with 10–15 kDa MW was used in the dialysis. Deionized (DI) water was used for all syntheses.

### 2.2. Synthesis of Mesoporous Silica Nanoparticles with Encapsulated Dyes

A previously reported procedure was used for the synthesis of UNSNP encapsulated with R6G and RB [26. Briefly, CTAB (0.69 g, 1.9 mmol), R6G, and RB were dissolved in 21.7 mL DI water at 90 °C. Another mixture of TEA (14.3 g, 96 mmol) and TEOS (1.71 g, 8.2 mmol) were mixed and kept at 90 °C for 3 h. The mixture of TEOS and TEA was then added to the mixture of dyes and CTAB. After mixing for 30 min, ETES (196 µL, 0.9 mmol) was added and stirred for 3 h. Three different R6G to RB molar ratios of 1:1, 1:0.1, and 1:0.01 were encapsulated in UNSNP, while the concentration of R6G was kept constant (0.073 g, 0.15 mmol), and the concentration of RB was changed. The membrane of MW 10–15 kDa was used to remove any excessive reagents.

### 2.3. Characterization

A Cary 60 UV-VIS (Agilent, Inc., Santa Clara, CA, USA) spectrophotometer was used to measure the absorbance spectra of the particles. Images and fluorescence spectra of single nanoparticles were obtained using WITec confocal Raman microscope (WITec, Inc., Ulm, Germany). The size distributions of nanoparticles were measured with a dynamic light scattering (DLS) technique (Zetasizer Nano ZS by Malvern, LTD., Malvern, UK) and Icon atomic force microscope (Bruker Inc, Santa Barbara, CA, USA). Fluorescence was measured using a Cary Eclipse fluorescent spectrometer (Agilent). The fluorescence lifetime of the encapsulated dye molecules was measured using the FLIM module of a laser scanning confocal Leica microscope (STELLARIS 8, Leica Microsystems Inc., Deerfield, IL, USA).

### 2.4. Simulation

**Figure 1:**
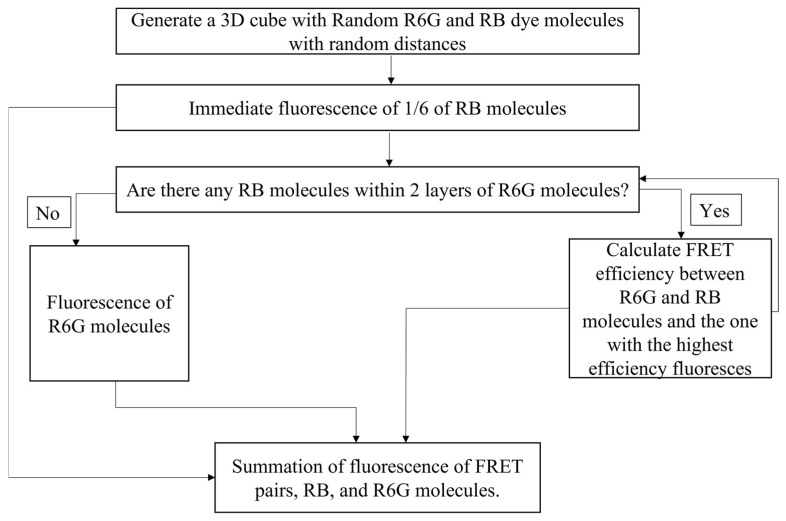
A flow chart of the steps involved in the simulation of fluorescence spectra of SiNPs encapsulated with two different dye molecules.

To simulate the fluorescence spectra in different nanoparticles, the following algorithm was used. Because the fluorescence spectra are defined by the individual dye molecules and their mutual positioning inside a nanoparticle, we built a simulation of the resulting fluorescence. The algorithm illustrated schematically in Figure 1 is as follows.

1. The nanoporous silica material of the nanoparticle is simulated with a cubic mesh, and the dye molecules are located at the knots of this mesh. To mimic a realistic situation, the size of each mesh component is “disturbed” by randomly generated size, assuming the normal distribution with the mean and dispersion equal to the average values of distances measured and presented in Table 1.
2. The numbers of R6G and RB dye molecules are measured with UV-VIS and presented in Table 1. The dye locations (in the mesh nodes) are randomly assigned in the Monte-Carlo manner.
3. The fluorescence emission is simulated with cycles. At the beginning, 1/6 of random RB dyes fluoresce before gaining any photons from R6G dyes. This number is chosen based on the absorption of RB dyes at the excitation wavelength (488 nm), which is 1/6 of R6G dyes.
4. At each cycle, R6G dyes (donors) look for the available RB dyes (acceptors) within 2 layers of the mesh as well as those located in the diagonal line. Following the calculation of the distances between the donors and acceptors, fluorescence resonance energy transfer (FRET) happens between those with the highest efficiency. If there are no RB dyes available, the relaxation of R6G dyes happens using their fluorescence.
5. Neither R6G nor RB dyes can absorb excitation energy when they are excited. They both become available after emitting photons (or for R6G, when transferring energy from R6G to RB through FRET).
6. During each cycle, each of R6G and RB molecules spends the same time in the excited state. This is based on direct measurements of their fluorescence lifetime (see the Results and Discussion section).
7. For simplicity, we consider each particle to be of cubic geometry.
8. 100 cycles are completed to simulate the spectrum of each particle.

The simulated fluorescence spectrum is produced by the following equation:

(1) $$\mathrm{FL}\left(\lambda \right)=\left(N_{R6G}-N_{\mathrm{FRET}}\right)\times {\mathrm{QY}}_{R6G}\times {\mathrm{Ab}}_{R6G}\times {\mathrm{FL}}_{R6G}\left(\lambda \right)+N_{\mathrm{FRET}}\times {\mathrm{Ab}}_{R6G}\times {\mathrm{QY}}_{\mathrm{RB}}\times {\;E}_{\mathrm{FRET}}\times {FL}_{RB}\left(\lambda \right)\;+\frac{1}{6}\times N_{\mathrm{RB}}\times {\mathrm{QY}}_{\mathrm{RB}}\times {\mathrm{Ab}}_{\mathrm{RB}}\times {FL}_{RB}\left(\lambda \right)\;$$

where $N_{R6G}$ and $N_{\mathrm{RB}}$ are the numbers of R6G and RB molecules inside each nanoparticle, respectively; $N_{\mathrm{FRET}}$ is the number of FRET pairs inside nanoparticles;${\;\mathrm{QY}}_{R6G}$ and ${\mathrm{QY}}_{\mathrm{RB}}$ are the quantum yield of R6G and RB; ${\mathrm{Ab}}_{R6G}$ and ${\mathrm{Ab}}_{\mathrm{RB}}$ are the absorbance of R6G and RB at the excitation wavelength, respectively; and $E_{\mathrm{FRET}}$ is the efficiency of FRET. $FL_{R6G}$ and $FL_{RB}$ are the fluorescence spectra of the corresponding dyes measured after encapsulation in the nanoporous silica. The values of the quantum yield of R6G and RB, as well as the Förster distance for calculating the efficiency of FRET, were measured and calculated as described in the previous study [26]. The number of R6G and RB dye molecules for each ratio were calculated using the method described in [20,24,27,29] (see the Supplementary Materials for detail). It should be noted that possible reabsorbance of the emitted fluorescence photons by other dye molecules was ignored in this study because the probability of such is much less than the absorbance of photons from the initial flux [29].

## 3. Results and Discussion

### 3.1. Particle Sizes

**Figure 2:**
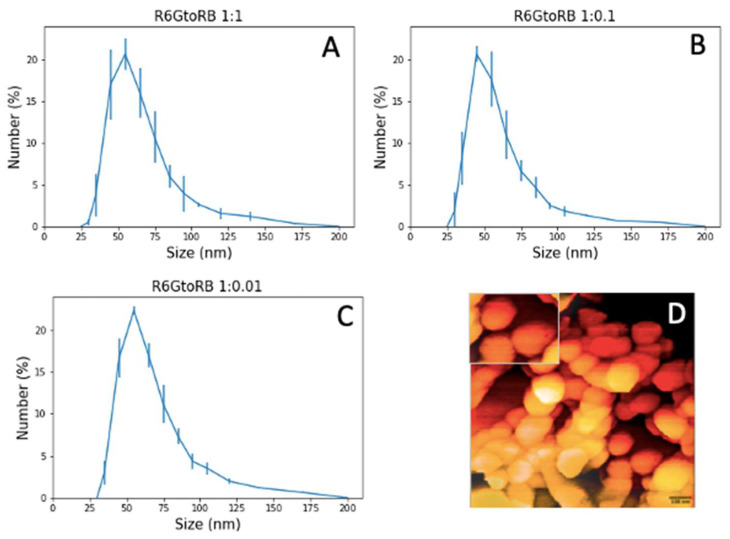
The results of dynamic light scattering (DLS) measurements of the particle size distribution at three different ratios of R6G to RB, including 1:1, 1:0.1, and 1:0.01, (**A**–**C**), respectively. (**D**) AFM image of nanoparticles with the scale bar of 100 (nm). A 500 × 350 nm insert clearly demonstrates the particle size.

Three different variants of nanoparticles were synthesized with the molar ratios of R6G to RB of 1:1, 1:0.1, and 1:0.0, as described in the Materials and Methods section. Figure 2A–C presents the results of DLS measurements of nanoparticle sizes. The size of UNSNP was measured three times using the DLS technique. The average size of UNSNP for R6G to RB ratios of 1:1, 1:0.1, and 1:0.01 were ~52 nm, 48 nm, and 52 nm, respectively. Thus, the particle size is very weakly dependent on the ratio of encapsulated dyes. The AFM image for R6G to RB ratio of 1:1 is shown in Figure 2D, which verifies the roundish geometry of the particles approximately the same size as measured with DLS.

### 3.2. Measurements of Fluorescence Spectra of Individual Nanoparticles

The measurements of fluorescence spectra from individual particles were performed using a confocal Raman microscope working in fluorescent mode. The particles were adsorbed to a glass slide and imaged while immersed in water. Each pixel in the image contains the recorded full luminescent spectrum. It is important to note that the image of a particle can contain several pixels. Therefore, we needed to ensure that there was no substantial spectral variation between the spectra at the edge versus the center of the particles. A representative image of the spectra is shown in supplementary Figure S2. One can see that there is no ambiguity in recording the spectra from different pixels associated with the particle.

The next important note about the fluorescence measurement of an individual particle is about the possible particle aggregates. Although we do not observe a substantial number of aggregates in dynamic light scattering measurements, it is hard to expect no aggregates when particles are adsorbed on the glass slide. Although we do not expect a change in the fluorescence spectra of aggregates, it may mistakenly yield effective averaging. This can produce artificially low fluctuations of the fluorescence ratio of individual nanoparticles. For example, if we deal with a cluster of five particles, a single measurement would be equivalent to the measurements of the average of five particles simultaneously. Fortunately, it can easily be identified by their brightness. For example, the particle size distributions shown in Figure 2 range between 40 nm and 70 nm (the semi-width of the distribution). Assuming a plausible proportionality between the particle volume and its fluorescence intensity, we can find the range of intensities for a single nanoparticle. The average size of a single particle based on Figure 2 is approximately 55 nm. Assuming that this particle has an intensity of 1000 (au, arbitrary units), one can find that the range of intensities for single nanoparticles should be between ~400 and 2100 au. Particles with these relative intensities were used in the measurements.

**Figure 3:**
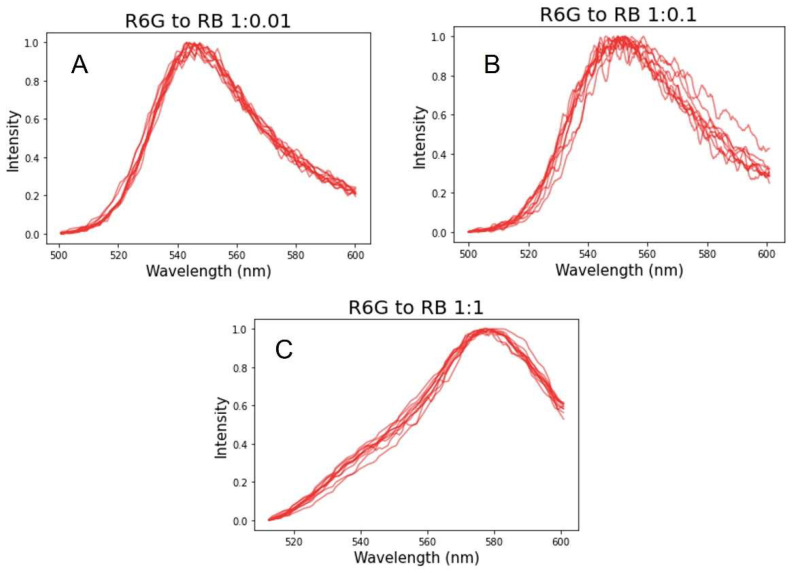
Fluorescence spectra of synthesized SiNPs with R6G to RB ratios of (**A**) 1:0.01, (**B**) 1:0.1, and (**C**) 1:1. All spectra are normalized to have their maxima at 1.

The results of the measurements of fluorescence spectra come from ten individual nanoparticles as shown in Figure 3 for each concentration of the dyes. One can see rather consistent spectra. To see it better, all spectra are normalized to have their maxima at 1.

### 3.3. Simulations of Fluorescence Spectra of Individual Nanoparticles and Comparisons with the Observed Spectrum

Since the number of measured particles is limited, it is important to see if these results are representative. We conducted simulations of the fluorescence spectra as described in the Materials and Methods section. As was mentioned in the description of the simulations, we needed to know the fluorescence lifetime of the encapsulated dyes. According to the previous study, the fluorescence lifetime of free R6G molecules and free RB molecules are 4.08 ns and 1.68 ns, respectively, in water [38]. However, the fluorescence lifetime of the donor decreases in the presence of an acceptor [39]. Furthermore, the environment surrounding the dye is quite different from water. Each dye molecule is surrounded by alkane chains of template surfactant molecules [29]. Thus, the lifetime might be substantially different from the one reported in the literature. Here, we performed FLIM measurements with a Leica scanning confocal microscope, which showed the lifetime of R6G molecules is 1.5 ± 0.3 ns, whereas RB is 1.5 ± 0.9 ns. Therefore, R6G and RB molecules spend the same time in the excited state, which was the observation implemented in the simulation algorithm.

To find the number of dye molecules per particle needed for simulation, we used UV-VIS spectroscopy to measure absorbance. To find the total volume of the synthesized particles that produce the measured absorbance, we measured particle concentrations. Following the method described in [20,24,27,29], the particle concentration was found with a direct weighting of synthesized UNSNP (see Supplementary Materials for details). Table 1 shows the result of the calculation of the number of dye molecules and the distance between them in each particle for each type of synthesized nanoparticle. The average distance was found by assuming a uniform distribution of the dye inside the particles. The average number of dye molecules was calculated based on absorbance and the known extinction coefficient of dye molecules, resulting in their concentration.

The number of UNSNP was calculated based on the weight, density, and volume of UNSNP. Dividing the number of dyes per volume by the number of UNSNP per volume resulted in the number of dyes per single particle. The distance between dye molecules was calculated based on the total number of dye molecules in the average volume of a single UNSNP.

It should be noted that the absolute intensity of the fluorescence spectrum is not that important for ratiometric sensing. The variations of the readings in ratiometric sensing are defined mainly by the ratio between the sensing and calibrating dyes. In our simulations, we assumed a homogeneous mix of both dyes, and the ratio between them can be found with UV-VIS spectroscopy with rather good precision. The error in the measurements of the number of each dye molecule shown in Table 1 comes from the error of the mass of the particles, i.e., the number of the particles in the aqueous dispersion of the particles used for the UV-VIS measurements.

FRET efficiency was calculated based on the equation: $\mathrm{Efficiency}={\;R}_{0}{}^{6}/\left(R_{0}{}^{6}+r^{6}\right),$ where $R_{0}$ is the Förster distance and r is the distance between dye molecules. R_0_ was calculated to be 8.79 nm considering the emission spectrum of R6G dye as a donor and the absorbance spectrum of RB as an acceptor [26]. In our simulations, dye molecules were located in the knots of a random-sized mesh. Therefore, the distance between dye molecules and the efficiency of FRET needed to be calculated for each FRET pair separately.

The quantum yield of R6G and RB dyes after encapsulation was taken from the literature to be 0.95 and 0.31, respectively [29]. The minimum distance between dyes was treated as a random parameter from a normal distribution with an average which was calculated with the assumption of a homogeneous distribution of dyes across the entire particle. The random allocation of the dye molecules inside a silica particle was created with the random assignment of all dyes of the particle to vacant positions in the silica mesh. After all these steps, the fluorescence spectrum was fully defined by Equation (1).

**Figure 4:**
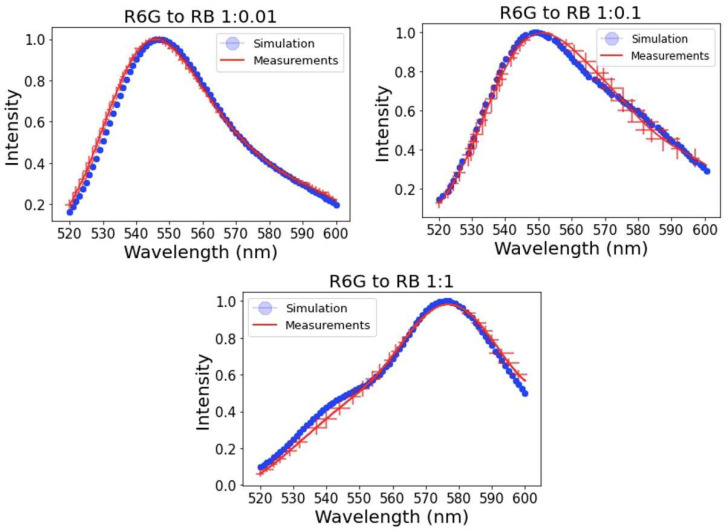
Fluorescence spectra of UNSNP with R6G to RB ratio of 1:0.01, 1:0.1, and 1:1. The blue line is the average fluorescence spectra from the simulation, and the red line is the fluorescence spectra measured on 10 individual nanoparticles. One standard deviation is shown for the measurements of intensity (the vertical line of the cross) and the averaged region on the wavelengths (the horizontal line of the cross).

Figure 4 shows an explicit comparison of the results of simulations of the fluorescence spectra of hundred particles. The random choice of the mesh and the location of the dye molecules was repeated 100 times. In the same figure, we plot the average and one standard deviation of the experimental measurements of fluorescence spectra shown in Figure 3. One can see a rather good agreement. This confirms that the measured fluorescence spectra of individual nanoparticles are representative.

Although the variations of the spectra seen in Figure 3 are quite small, it is instructional to transfer the observed fluctuations into something measurable using these particles as sensors. Since these particles were previously used for the temperature measurements, we elected to translate the observed fluctuations in terms of uncertainty of measuring temperature. The temperature dependence of fluorescence spectral ratio was measured in a similar way as described previously in [11]. The connection found between the temperature and ratio was also linear and can be presented as T=11.48× R+20.75, where T is the temperature in Celsius, and R is the ratio of fluorescence intensity of R6G to RB. Consequently, the fluctuation in the ratio is transferred into temperature as follows δT=11.475× δR. It provides the fluctuations in the measurement of temperature of 0.4 °C when using one particle and 0.1 °C when using the results of averaging over five particles (signal collection time for each spectrum was 30 ms). Interestingly, the random generation of 10 fluorescent particles in our simulations produced rather similar fluctuations of temperature measurements, specifically, 0.3 °C and 0.1 °C for one and five particles, respectively. Further investigation of these particles as nanothermometers is beyond the scope of this work and will be explored in future works.

## 4. Conclusions

Here, we addressed a fundamental concern of making single nanoparticle ratiometric sensors: the uncontrollable ratio between different encapsulated fluorescent dye molecules. It should lead to a variation of fluorescence spectra between different particles [1,2,3,4]. The majority of ratiometric sensors deal with two fluorescent dyes (one as a reference and the other dye as the sensing one) [5,7,9,11]. Encapsulation of both dyes within one nanoparticle is technically impossible at precisely the same dye ratio. Therefore, there is a fundamental limitation to the accuracy of such sensors. To address this concern, we used an example of ultrabright nanoporous silica nanoparticles, which have two encapsulated dyes and are typically used for the measurements of temperature. The consideration of these particular particles has practical importance because ultra-high fluorescence brightness allows reliable imaging of individual nanoparticles. Direct measurements of the fluorescence spectra coming from these single particles showed rather small variations in the spectrum. Recognizing that any finite number of measurements can be representative only in a limited way, we simulated the fluorescent spectra of the particles, in which we randomized the dye molecule locations and distances between them within each nanoparticle. All the parameters needed for the simulation were directly measured. Based on the results described above, one can see that the measurements of the fluorescence spectrum of individual nanoparticles are in very good agreement with the simulation results. Therefore, we can claim that the directly measured variations of the fluorescent spectra are representative.

Translating the fluctuation of the spectrum into a measured value such as temperature, one can see that individual particles can indeed be used for a reasonable measurement of temperature. For example, just a single particle can be used to measure the temperature with an error of 0.4 °C when the spectrum is collected for 30 ms. When the measurements are performed using several particles simultaneously, the accuracy substantially increases. According to the ergodic hypothesis, a similar increase in accuracy can be attained with a longer accumulation of the signal in time. A particular application of these particles to the measurement of temperature will be studied in our future papers.

In conclusion, we demonstrated that ultrabright fluorescent silica nanoparticles could be used as effective ratiometric sensors when two different dyes are encapsulated inside a nanoporous silica matrix. Although one cannot control the ratio of different encapsulated dye molecules, the differences in the ratio and the final spectrum are rather small, as seen in both direct measurements and simulations. This is presumably the result of thermodynamic averaging of the number of dye molecules and their positions inside each particle, as well as a result of a relatively large number of dye molecules encapsulated within each particle. It is an important result for the development of single nanoparticle nanosensors because it shows that concern about the uncontrolled locations of encapsulated dyes inside each nanoparticle is not a major obstacle to the development of nanoparticle-based fluorescent ratiometric nanosensors.

## Figures and Tables

**Table 1:** Numbers of Dye Molecules in the Synthesized Nanoparticles.

Ratio of R6G to RB	Number of R6G	Number of RB	Distance between Dye Molecules (nm)
1:1	1267 ± 172	1525 ± 198	3.04 ± 0.14
1:0.1	1274 ± 40	510 ± 44	3.53 ± 0.05
1:0.01	1341 ± 72	86 ± 11	3.80 ± 0.07

## Data Availability

The data supporting the findings of this study are available from the corresponding author upon reasonable request.

## Supplementary Materials

The following supporting information can be downloaded at: https://www.mdpi.com/article/10.3390/s23073471/s1, Figure S1: The absorbance spectrum of ultrabright nanoporous silica nanoparticles with R6G to RB ratio of (A) 1:0.01, (B) 1:0.1, and (C) 1:1; Figure S2: Fluorescence spectra recorded at different locations/pixels corresponding to the nanoparticle of interest; Section S1: Calculation of the number of R6G and RB molecules in each nanoparticle.
