# Supplementary material for: Towards the Use of Individual Fluorescent Nanoparticles as Ratiometric Sensors: Spectral Robustness of Ultrabright Nanoporous Silica Nanoparticles
Source: Sensors (Basel). 2023 Mar 26;23(7):3471. doi: 10.3390/s23073471 (PMC10098630; doi:10.3390/s23073471)
Supplement: Supplementary file 1 [file sensors-23-03471-s001.zip › sensors-2306389-supplementary.pdf]

Supplementary materials
for

Towards the Use of Individual Fluorescent Nanoparticles as Ratiometric Sensors: Spectral Robustness of Ultrabright Nanoporous Silica Nanoparticles

Mahshid Iraniparast[1], Berney Peng[1,†], Igor Sokolov[1,2,*]

[1] Department of Mechanical Engineering, Tufts University, Medford, MA 02155, USA

[2] Department of Physics, Tufts University, Medford, MA 02155, USA

[†] Present address: MacDonald Medical Research Laboratories, UCLA, CA 90095, USA

* Correspondence: igor.sokolov@tufts.edu.

1. Calculation of the number of R6G and RB molecules in each nanoparticle

The mass of particles per unit volume was calculated for each ratio by drying nanoparticles in the oven at 60 °C for 12 hours. The measurement was repeated three times, and the masses were 2.36±0.05 mg/mL, 2.25±0.03 mg/mL, and 2.1±0.07 mg/mL for ratios of 1:0.01, 1:0.1, and 1:1, respectively. According to the mass density of porous silica (1.6 g/cm^3) and the size of the particles for each ratio (Figure 2), the total number of particles in the stock solution was calculated to be 4.4x10^{11} /mL, 1.23x10^{11} /mL, and 1.15x10^{11} /mL for ratios of 1:0.01, 1:0.1, and 1:1, respectively. The known volume of particles from the stock solution was added to the water and measured by UV-VIS absorbance. Finally, the number of encapsulated dye molecules for each ratio was calculated using the Beer–Lambert law according to the known extinction coefficients of R6G and RB (1.0x10^5 L/mole/cm at 525 nm for R6G and 1.2x10^5

L/mole/cm at 550 nm for RB dye). The results are shown in Table 1 of the main manuscript. The absorbance spectra of SiNPs with ratios of 1:0.01, 1:0.1, and 1:1 are shown in Figure S1A-C.

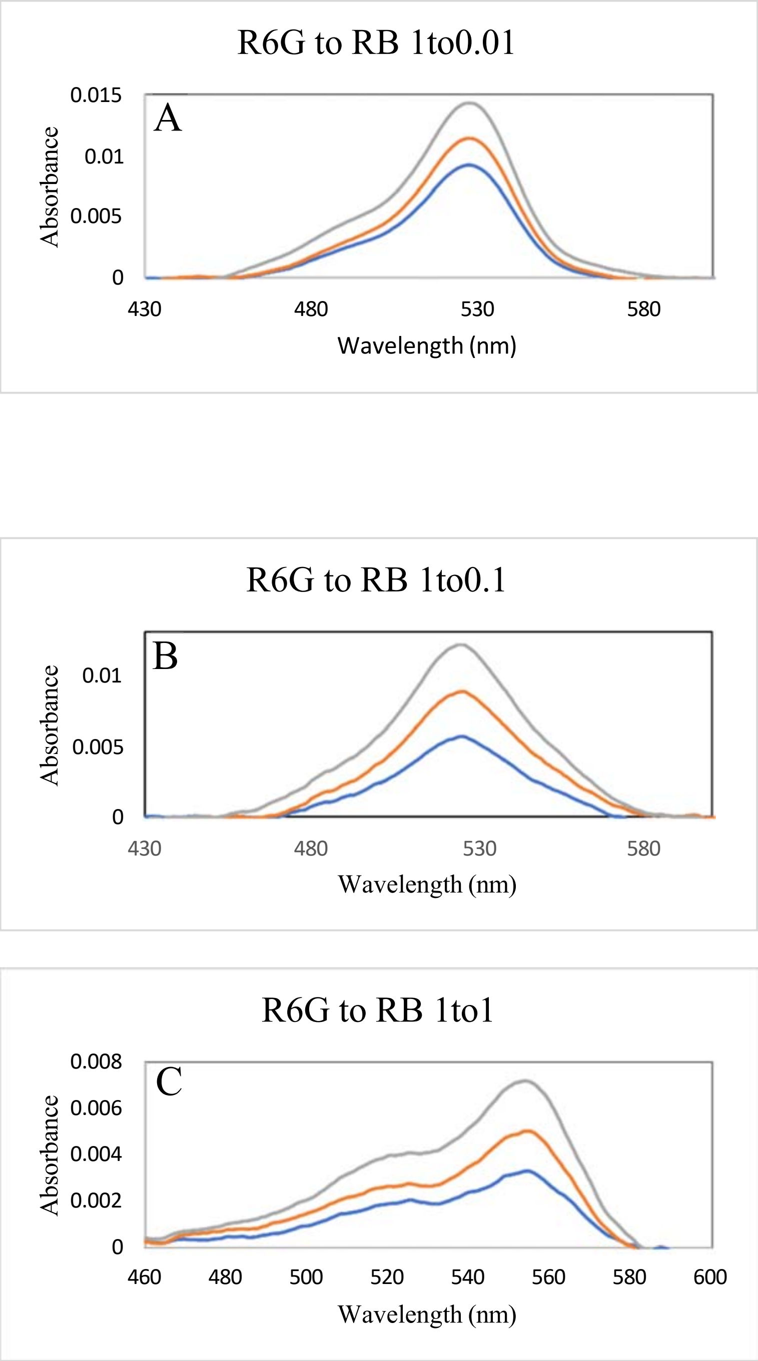

Figure S1. The absorbance spectrum of ultrabright nanoporous silica nanoparticles with R6G to RB ratio of (A) 1:0.01, (B) 1:0.1, and (C) 1:1.

2. Fluorescence spectra of different spots of a nanoparticle

The fluorescence spectra at the different locations of the particle/cluster were measured, shown in Figure S2. There were no substantial differences in the spectra.

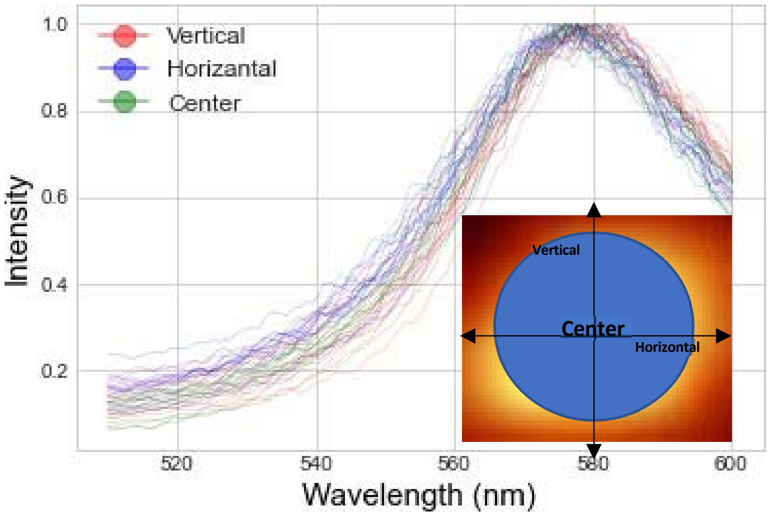

Figure S2. Fluorescence spectra recorded at different locations/pixels corresponding to the nanoparticle of interest.
